# A Cardiac Deep Learning Model (CDLM) to Predict and Identify the Risk Factor of Congenital Heart Disease

**DOI:** 10.3390/diagnostics13132195

**Published:** 2023-06-28

**Authors:** Prabu Pachiyannan, Musleh Alsulami, Deafallah Alsadie, Abdul Khader Jilani Saudagar, Mohammed AlKhathami, Ramesh Chandra Poonia

**Affiliations:** 1 Department of Computer Science, CHRIST, Bangalore 560029, India; prabu.p@christuniversity.in; 2 Information Systems Department, Umm Al-Qura University, Makkah 21961, Saudi Arabia; dbsadie@uqu.edu.sa; 3 Information Systems Department, Imam Mohammad Ibn Saud Islamic University (IMSIU), Riyadh 11432, Saudi Arabia; aksaudagar@imamu.edu.sa (A.K.J.S.); maalkhathami@imamu.edu.sa (M.A.)

**Keywords:** newborn, mortality, congenital heart disease, machine learning, heart disease, healthcare

## Abstract

Congenital heart disease (CHD) is a critical global public health concern, particularly when it comes to newborn mortality. Low- and middle-income countries face the highest mortality rates due to limited resources and inadequate healthcare access. To address this pressing issue, machine learning presents an opportunity to develop accurate predictive models that can assess the risk of death from CHD. These models can empower healthcare professionals by identifying high-risk infants and enabling appropriate care. Additionally, machine learning can uncover patterns in the risk factors associated with CHD mortality, leading to targeted interventions that prevent or reduce mortality among vulnerable newborns. This paper proposes an innovative machine learning approach to minimize newborn mortality related to CHD. By analyzing data from infants diagnosed with CHD, the model identifies key risk factors contributing to mortality. Armed with this knowledge, healthcare providers can devise customized interventions, including intensified care for high-risk infants and early detection and treatment strategies. The proposed diagnostic model utilizes maternal clinical history and fetal health information to accurately predict the condition of newborns affected by CHD. The results are highly promising, with the proposed Cardiac Deep Learning Model (CDLM) achieving remarkable performance metrics, including a sensitivity of 91.74%, specificity of 92.65%, positive predictive value of 90.85%, negative predictive value of 55.62%, and a miss rate of 91.03%. This research aims to make a significant impact by equipping healthcare professionals with powerful tools to combat CHD-related newborn mortality, ultimately saving lives and improving healthcare outcomes worldwide.

## 1. Introduction

Every year, millions of babies are born with congenital heart disease (CHD), a condition in which the heart does not develop properly [1]. CHD is the leading cause of newborn mortality, accounting for nearly one-third of all infant deaths. There are many different types of CHD, ranging from mild to severe. The most common type, ventricular septal defect (VSD), affects the septum, which is the wall separating the right and left ventricles [2,3,4]. VSDs can be small, allowing blood to flow freely between the ventricles, or large, causing the ventricles to work harder and the heart to pump less effectively [5]. If a child is born with heart disease, the condition is called congenital. Statistics show that only about 1% of babies are born with this disease. Heart disease is widespread [6], and it depends on the lifestyle the mother leads while carrying the fetus. The baby’s health is determined in the first months of pregnancy [7]. Suppose there is an expectant mother during this period. In that case, the risk of having a child with a heart defect increases significantly: if one notices the signs of heart disease in children early and starts treatment, there is a chance of full recovery and normal functioning [8,9].

Conversely, if the problem is detected late, irreversible changes in the heart muscle structure occur, and emergency surgery is required [10]. Sometimes, people live without a kidney, half a stomach, and a gall bladder. However, it is impossible to imagine a person living without a heart: after this organ stops its work, in a few minutes, the life in the body dies completely and irreversibly [11,12]. This is why diagnosing “heart disease” in a child is so terrifying for parents. If we avoid medical nuances, this disease is associated with the failure of the heart valves, which leads to the gradual failure of the organ [13]. This problem is the most common cause of heart disease but is far from the only disease [14]. In addition, there are cases when the disease develops due to incorrect structure. Different treatments for congenital heart defects include open-heart surgery in the cardiac catheterization lab [15]. Some babies may also need medicines to control their heart rate or blood flow. Treatment of complex congenital heart defects may require certain surgery [16]. Critical congenital heart defects (CHDs) need several catheter surgical procedures and open-heart surgeries to repair the problems [17]. A combination of catheter and open-heart surgery is required for some babies. Surgery or other procedures may be needed for other newborns before they can go home from the hospital [18,19]. Only a few of four babies need surgery for critical CHDs in the first few years of life. These children typically need to take lifelong medication after their treatment [20].

Surgery and cardiac catheterization are the two main treatments for congenital heart surgery for newborns. In some cases, repairing a simple heart defect may be possible through cardiac catheterization in a special lab, but more complex defects may require open-heart surgery [21]. Atrial septal defect, ventricular septal defect, and patent ductus arteriosus are some of the most effective forms of CHD that require surgery [22]. Different techniques are used to repair these defects, such as using different devices to reduce the effects of air or to close up the patent ductus arteriosus [23]. Congenital heart surgery for newborns can be complex and require specialized care. Children with complex congenital heart defects or CHD may require multiple surgeries over the first few years of life or even a heart transplant [24,25]. For more straightforward heart problems, such as very mild stenosis or atrial septal defect, imaging studies such as an echocardiogram can diagnose the issue. Hypoplastic left heart syndrome (HLHS) is one of the most severe forms of CHD, and these children may require several surgeries or a transplant to survive [26,27]. Sometimes these surgeries fail, and the child must receive a heart transplant to live. Congenital heart surgery for newborns is complex and delicate [28].

Congenital heart surgery treats many cardiac defects that can improve blood flow in the heart and lungs, condition the heart rate and prevent heart failure [29]. Some congenital heart defects, like ventricular septal defects or Fallot’s other complex anomalies, require repair with cardiopulmonary bypass or stopping the baby’s heart to control it [30]. Diagnosing a disease in a child is a mandatory study at the stage of intrauterine development. For every woman at 14 weeks of pregnancy, the supervising doctor orders an ultrasound of the heart. Ultrasound is the primary method for diagnosing CHD and PPD [31]. Diagnostics using ultrasound help visualize the structural compartments of the heart, as well as calculate pressure and other parameters [32]. If heart failure is suspected, the cardiologist recommends additional diagnostic methods. Electrocardiography is used for detecting congenital and acquired defects at any age [33]. It also corrects arrhythmias, electrical axis displacements, and conduction system disorders. ECG is included in mandatory examinations for one-month-old babies [34]. X-rays are used to determine the current condition of the chest and heart.

Specialists choose a method of treatment taking into account the type of pathology, stage of development and complexity in each case [35]. The condition and age of the sick child play an essential role in choosing the optimal treatment option. Conservative treatment often includes diet, general hygiene and physical exercises [36]. Sick children are advised to consume protein-rich foods, limit water and salt intake, and avoid food before bed. Children should also perform special exercises to help train the heart muscle [37]. Clinical symptoms of PBS are often determined only with diagnostic help, taking into account the type of defect, its degree of severity and development. Symptoms appear depending on the localization parameters and the number of affected valves [38]. Also, the symptoms of acquired deficiency in a child may differ depending on the functional form of the pathology. Symptoms of the pathology are not always detected at the initial stage of the development of the pathology. Most often, the symptoms of the disease in a child appear after a few months or years [39].

In newborns, heart disorder symptoms differ depending on the presence of a specific anomaly, but these symptoms can be generalized. The main contribution of this research paper is as follows:

- Planning for the baby’s treatment before they are born is essential. If the child has been diagnosed with a congenital heart defect, the doctor plans the best treatment for their condition based on the machine learning approach.
- The treatments may include open-heart surgery or a heart transplant. In some cases, babies may need a catheter procedure instead of open-heart surgery if the defect is not too severe. Catheter interventions are often used for mild heart defects. However, it is required to predict severe heart problems, and open-heart surgery is usually recommended with the help of a machine learning approach.
- After surgery, monitoring the health and seeing a CHD specialist is essential to ensure the defect does not worsen or that other health problems do not develop. An artery intervention may be necessary in the long term. These factors should be monitored with the help of a machine-learning model [40]

Preterm neonates with CHD face ongoing risks, and their neurodevelopmental outcomes are poorly understood. In this work, the researchers examine preterm birth rates in CHD infants, emphasize their complex medical needs, and stress the importance of evaluating outcomes beyond survival. They explore shared mechanisms of neurodevelopmental impairment in CHD and prematurity, proposing future directions for improved outcomes [41]. A total of 12,926,083 individuals (ages 3–18) from 86 studies had a CHD prevalence of 4.69 per 1000 children. CHD prevalence decreased over time from 6.19 to 3.30 per 1000 children. High-altitude areas had a higher CHD prevalence (OR 2.84) with patent ductus arteriosus being the common subtype. Low-altitude areas had an OR of 1.31 with atrial septal defects as the predominant subtype, which highlights the importance of prioritizing high-altitude and economically underdeveloped areas in the allocation of medical resources and healthcare for women and children in China [42].

Congenital heart defects affect many people worldwide, ranging from mild to severe forms of the condition. It is essential for individuals with CHD problems to seek medical care from a specialist as soon as possible in order to prevent long-term complications and reduce the likelihood of developing other health issues.

## 2. Literature Review

A pathological classification of a routine nature divides CHD conditions into categories depending on their impact on the child’s development. Unfortunately, in recent years, the incidence of CHD in infants and premature infants has been increasing, and the anatomical features of the disease are changing.

Edupuganti, M. et al. [21] discussed that persistent heart structure changes characterize acquired defects in children and adolescents. The initial change is carried out during birth, causing a disorder in the functioning of the heart (Eltahir, M.M. et al. [22]). In clinical practice, acquired heart defects are classified in different ways. The stimulation of the skin triggers the white type of CHD. It is characterized by the release of blood from an arterial circulation into a venous one. White CHD is determined by open duct arteriosus and isolated lesions of the aorta and septum. Katarya, R. et al. [23] discussed that blue-type defects are visually distinguished by the cyanosis of the skin (persistent cyanosis) transfer of large vessels. It is caused by a process where the aorta leaves the right ventricle, and the pulmonary artery, on the contrary, proceeds from the left. Nadakinamani, R.G. et al. [24] expressed that the Fallot triad combines several disorders like narrowing the pulmonary artery, ventricular septal defect, and aortic and right ventricular defects. These include the right vena cava atresia, pulmonary artery, aorta, etc.

Tan, W. et al. [25] reported that Fetal echocardiography is a non-invasive method for diagnosing CHDs in fetuses. In recent years, machine learning methods have been developed to create predictive models of newborn mortality associated with this condition. The machine learning approach using artificial neural networks has to minimize the risk of death due to CHD. Hussain, L. et al. [26] expressed that the ANN prediction model was trained on medical data sets and tested for accuracy in predicting the outcome of newborns suffering from CHD. Nguyen BP further developed this approach by adding more machine learning techniques such as genetic algorithms, Bayesian networks and support vector machines to improve the accuracy of the predictive models. Al Ahdal, A. et al. [27] reported that the preliminary computational results using these machine learning approaches showed promising outcomes for neonatal and adult patients with hematologic malignancies. The diagnosis model enabled accurate disease diagnosis by leveraging data from history using machine learning techniques, leading to more accurate predictions of patients’ prognosis and treatment options. Dritsas, E. et al. [28] expressed that screening tools and predictive models can be used to identify risk groups for newborns suffering from CHD. These tools allow for early detection, helping identify those at higher risk and providing appropriate care. Ng, W. et al. [29] discussed that machine learning is also being used to develop a risk index which can help in the present analysis of pregnancy and predict the possibility of a newborn having CHD. This approach uses data from the mother’s health history, ultrasound scans, and other factors to assess the risks associated with pregnancy accurately.

Balakrishnan, M. et al. [30] expressed that machine learning algorithms such as logistic regression and decision trees can be used to analyze the data collected from maternal laboratory tests, clinical laboratory data, and other studies predicting CHD. The results of these predictive tools can be used to identify potential risk factors and create a set of results predictors. Williams, R. et al. [31] expressed that applying CHD prediction using machine learning techniques such as supervised learning, it is possible to develop predictive models that can accurately predict CHD in newborns. This approach effectively reduced the mortality rate of newborns suffering from CHD. Ravi, R. et al. [32] discussed that by using the data obtained through laboratory tests combined with machine learning algorithms such as logistic regression and decision trees, doctors can create accurate predictive models to help them determine the likelihood of a baby being born with CHD. Pei, Y. et al. [33] expressed that doctors can be better prepared for the postoperative complications that may arise. Additionally, integrated patient data from various sources can be used to create efficient machine learning models using ML algorithms to identify at-risk newborns before birth. It can help reduce the intraoperative time needed to treat CHD, thus minimizing the suffering of newborns with this congenital disability. Shishah, W. et al. [34] discussed that by combining various models with clinical data collected during pregnancy and delivery, doctors can develop more accurate models that could potentially prevent many congenital disabilities in newborns. The machine learning classification approach can identify and diagnose at-risk infants quickly, allowing doctors to take preventative measures early on. Iscra, K. et al. [35] expressed that machine learning technology has been utilized to develop predictive models for diagnosing newborns with CHD [43]. These models have been applied to large datasets of neonatal ICU admissions and showed promising results in terms of accuracy and speed of diagnostics [44]. It is particularly beneficial for newborns suffering from CHD, as early detection allows for prompt repair or intervention, which can help improve the chances of survival. The following Table 1 demonstrates the comparative analysis of the related works.

### 2.1. Research Gap

Congenital heart disease (CHD) poses a substantial risk to newborns, resulting in mortality. CHD refers to heart conditions present at birth which can lead to heart attacks and blood pressure complications. If left untreated, the consequences can be fatal. Treatment approaches for CHD encompass surgical procedures, medication, or a combination of both. Detection of CHD often occurs after birth, as symptoms may be mild and become apparent during periods of stress, such as viral illnesses. Addressing CHD presents challenges due to the intricate nature of the heart and the associated risks involved in corrective surgeries. Moreover, treating CHD becomes even more complex when additional health problems are present in affected infants.

### 2.2. Reseach Contribution and Novelty of the Work

This research study brings forth significant contributions and novel approaches to address the vital issues surrounding congenital heart disease (CHD) and its impact on newborn mortality. The researchers have made noteworthy strides in the following areas, highlighting the uniqueness of research.

Prenatal care and early detection: The study emphasizes the fundamental importance of prenatal care and early detection in improving outcomes for CHD. By shedding light on the significance of better access to quality medical care and advancements in surgical techniques, the researchers provide valuable insights for enhancing healthcare practices. Furthermore, their recognition of the need for support and resources to assist families affected by CHD showcases a comprehensive and holistic approach to addressing the challenges faced by these individuals.

Deep learning (DL) prediction for accurate risk assessment: Researchers made a distinctive contribution by harnessing the power of DL prediction, which outperforms other existing methods in terms of accuracy. This breakthrough holds immense promise for the field. Through the utilization of DL prediction, healthcare professionals can precisely identify high-risk patients and tailor treatment strategies accordingly. This novel approach ensures precise risk assessment and enables personalized care interventions, ultimately leading to improved patient outcomes and optimized healthcare delivery.

Monitoring and prediction in diverse settings: The study illuminates the researchers’ pioneering work in utilizing DL prediction for monitoring patient progress and detecting early signs of deterioration. Moreover, their demonstration of the effectiveness of DL prediction in various healthcare settings, including those with limited resources, is a remarkable achievement. This breakthrough has far-reaching implications for healthcare providers, empowering them to effectively track patient health and predict outcomes, thereby enhancing patient care and outcomes across diverse healthcare environments.

The unique contributions of this research lie in its innovative approaches to tackle the challenges associated with CHD. By underscoring the importance of prenatal care, introducing DL prediction for accurate risk assessment, and highlighting the applicability of this approach in diverse healthcare settings, the researchers have made significant advancements in the field. These contributions hold the potential to revolutionize CHD management, save lives, and improve healthcare outcomes for newborns affected by this condition.

## 3. Methodology

**Figure 1:**
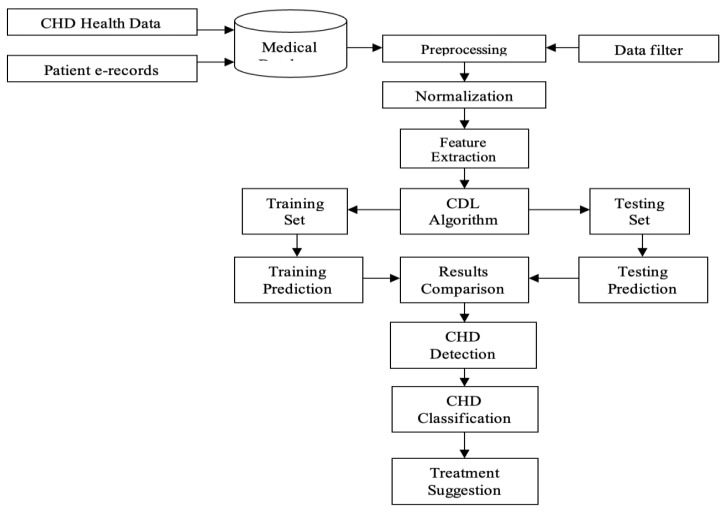
Block diagram of the proposed model.

The pathology can usually be diagnosed in children through routine examinations. A pediatrician who listens for extraneous sounds during auscultation of the heart makes a referral to a pediatric cardiologist. The specialist prescribes the necessary tests and assigns an accurate diagnosis. If a congenital heart defect is suspected during pregnancy, fetal echocardiography is performed—an ultrasound examination of the fetus in the womb. The proposed block diagram as shown in the following Figure 1.

The heart’s structure can be reasonably studied as early as ten weeks. Dilated echocardiography is performed in at-risk mothers. High-quality diagnostics can detect 60–80% of CHD before birth. In addition to technical methods, the diagnosis of heart defects is based on four required methods:

- Examination
- Rhythm (tapping)
- Palpation (probe)
- Auscultation (listening)

Heart defects are the most common cause of death in children under one year of age. That is why learning about the disease and its symptoms is essential. After all, a timely visit to a specialist can save a child’s life. Perhaps the partner will not be confirmed, and the parents’ fears will be in vain, but when it comes to the child’s health, it is better to play safe.

### 3.1. Symptom Dataset

The dataset is listed in Ref. [45]; it is an open-source dataset. The dataset reports the following: Systolic pressure is reduced, and diastolic pressure is normal or increased. With aortic insufficiency, there are no complaints during compensation; sometimes, tachycardia and a pulse behind the sternum are observed. In the stage of degeneration, angina pectoris occurs in the chest, in which nitroglycerin does not help sufficiently, and persistent symptoms like dizziness, fainting, shortness of breath (at first with exertion, then at rest), oedema, heavy or painful feeling under the ribs to the right are reported. Examination revealed pulsation of the pallor, peripheral arteries, a rhythmic change in skin colour with light pressure under the nails and lips, and head shaking synchronous with the pulse. The pulse is accelerated and high. The systolic and pulse pressure are increased, and diastolic pressure is decreased.

### 3.2. Preprocessing

Acquired heart disease in a child develops for many reasons. This disease affects the heart valves, in which granulomas form in the stroma. In 75% of cases, rheumatic endocarditis is responsible for rheumatic development. Connective tissue diseases spread. Lupus erythematosus, scleroderma, dermatomyositis and other pathologies often cause kidney and heart problems. Any powerful blows to the chest area can cause the development of a defect. After operations already performed on the heart, for example, valvotomy, complications can trigger the defect’s development. Atherosclerosis is a chronic disease of the arteries and blood vessels in which atherosclerotic plaques begin to form on their walls. Rarely, atherosclerosis also causes changes in the work and structure of the heart. This list shows that if a child develops a heart defect, the reasons for this are very diverse, but it is essential to at least detect them so that the prescribed treatment is efficient and more effective. The initial dataset used for conventional heart disease prediction contained a sample size of 407 patients and included 79 features. However, for the purpose of this study, the sample size was reduced to 369 patients. The study focused on nine specific features, which encompassed the monitored parameters relevant to heart disease prediction. These parameters included sex, blood pressure, resting electrocardiogram (ECG), maximum heart rate, birth weight, weight at 4 weeks, weight difference between birth and 4 weeks, blood oxygen saturation, and body temperature. In the dataset, systolic pressure is reduced, and diastolic pressure is normal or increased. With aortic insufficiency, there are no complaints during compensation; sometimes, tachycardia and a pulse behind the sternum are observed. The following preprocessing has been performed:

(a) Missing value imputed by KNN: KNN operates by computing the distance or similarity between data points to identify the most similar case within the dataset. This nearest neighbor is subsequently used to replace the missing value, offering an estimated value based on similar instances.

(b) Min_Max Normalization: In this method, each numerical feature value is converted into a new value based on the minimum and maximum values of that specific feature. By utilizing this approach, the feature values are rescaled to a common range, enabling effective normalization and facilitating comparisons across different value ranges.

(c) One Hot Encoding: The process of One Hot Encoding involves breaking down a categorical feature into a set of separate features based on the unique categories present. The number of new features created corresponds to the number of distinct cases in the original categorical feature. For each new feature, a value of 0 is assigned to indicate the absence of a specific category, while a value of 1 represents the presence of that category. This encoding technique enables categorical data to be represented in a format suitable for machine learning algorithms to process effectively.

### 3.3. Classification

Different deep learning algorithms can be used for classification tasks, and the choice of the best algorithm depends on the nature of the data and the desired outcome. When it comes to predicting newborn mortality in CHD, popular algorithms include support vector machines, decision trees, and artificial neural networks. Congenital malformation with blockages refers to a condition where there are difficulties in adequately draining blood from the ventricles. This condition can be classified into different types:

- Stenosis: This occurs when the aorta narrows in the region of the valve.
- Aortic consolidation: This refers to a pathology where the lumen in a specific area of the aorta is narrowed or completely closed.
- Pulmonary stenosis: This is a disorder in which the outflow tract of the right ventricle becomes narrow, obstructing blood flow into the pulmonary artery.

When choosing a deep learning algorithm for predicting newborn mortality in CHD, it is crucial to consider the specific data used for training and testing. For example, if the dataset is small, a more complex algorithm like support vector machines may not be necessary. Conversely, if the dataset is extensive or contains a significant amount of noise, a simpler algorithm such as a decision tree may struggle to learn the underlying patterns effectively. Additionally, the desired outcome of the classification plays a significant role. If the goal is to predict whether a newborn will die from CHD or not, a binary classification algorithm would suffice. However, if the aim is to predict the severity of the disease, a multi-class classification algorithm would be required. By carefully considering the dataset and desired outcome, one can compare different deep learning algorithms and select the most suitable one for the task.

### 3.4. Detection

For this task, we use a dataset of CHD cases from the US National Institutes of Health. This dataset includes information on demographics, risk factors, and outcomes for many patients. It builds a DL model to predict the probability of mortality for each patient in the dataset. It uses a multi-layer perception with input features, including demographics, risk factors, and outcomes. The DL model is trained and tested on the NIH dataset. We compare the results of our model to other predictive models for this task and discuss the implications of our findings. Symptoms of heart disease in children, observed by parents and pediatricians, are not yet the basis of a diagnosis. As mentioned above, it is also found in healthy children, so it cannot do without an ultrasound. An echocardiogram may show signs of left ventricular overload. In addition, a chest X-ray may be required, which shows changes in the heart and signs of deviation of the esophagus. After that, the matter of whether the child is sick or healthy can finally be discussed. Unfortunately, ECG cannot help diagnose heart disease in the early stages. The changes in the cardiogram are significant when the disease is already actively progressing.

## 4. Proposed Model

Machine learning is a powerful tool that can be used to build models that can accurately predict the risk of newborn mortality due to CHD. These models can identify babies at risk of developing complications and provide timely interventions to reduce mortality rates. The models can also be used to identify trends and patterns in health data to inform better public health strategies to reduce the incidence of CHD. Furthermore, machine learning can be used to develop personalized treatments for patients, improving the efficacy of treatments and reducing mortality rates. A machine learning approach to predict newborn mortality suffering from CHD would involve using a supervised learning algorithm. The structure of the proposed algorithm is shown in the following Algorithm 1. This algorithm is trained using historical data on the mortality rates of newborns with CHD, as well as other factors such as gestational age, birth weight, and maternal health history.
**Algorithm 1.** Cardiac deep learning algorithm// Get MRI image samples and set the range; Input: A_in_; Output: A_out_; // Segment the images; For each cluster pair (A_x_in_, A_y_in_)                    If min(A_x_in_, A_y_in_) × |Q_x_in_ − Q_y_in_|                    Then merge A_x_in_, A_y_in_ into A_z_; //Feature extraction;                    AZ_i_ = AZ_x_in_ + AZ_y_in_;                      Q_i_ = (AZ_x_in_ × Q_x_in_) + (AZ_y_in_ × Q_y_in_)/(AZ_x_in_ + AZ_y_in_); //Preprocessing of samples;                      Where A_in_ is the x × y matrix;                      For I = 1:x                      For j = 1:y                      If (A_in_(x,y) < 0                      A_in__s = 1;                      Else                      A_in__s = 0; End;

The algorithm is then used to make predictions of newborn mortality based on new data points, such as a newborn’s particular CHD diagnosis. By making predictions based on historical data, the algorithm can help doctors make more informed decisions about caring for a newborn with a CHD diagnosis. The algorithm provided is designed to process MRI image samples. It involves several key steps to transform the input data into a desired output. First, the images are segmented, combining certain clusters based on predefined conditions. Then, features are extracted from the clusters, producing new values for further analysis. The algorithm also includes a preprocessing step, where each element in the input matrix is evaluated and assigned a specific value based on certain conditions. This helps prepare the samples for subsequent stages. Overall, this algorithm offers a structured approach to analyzing MRI images, allowing for improved understanding and utilization of the data.

**Figure 2:**
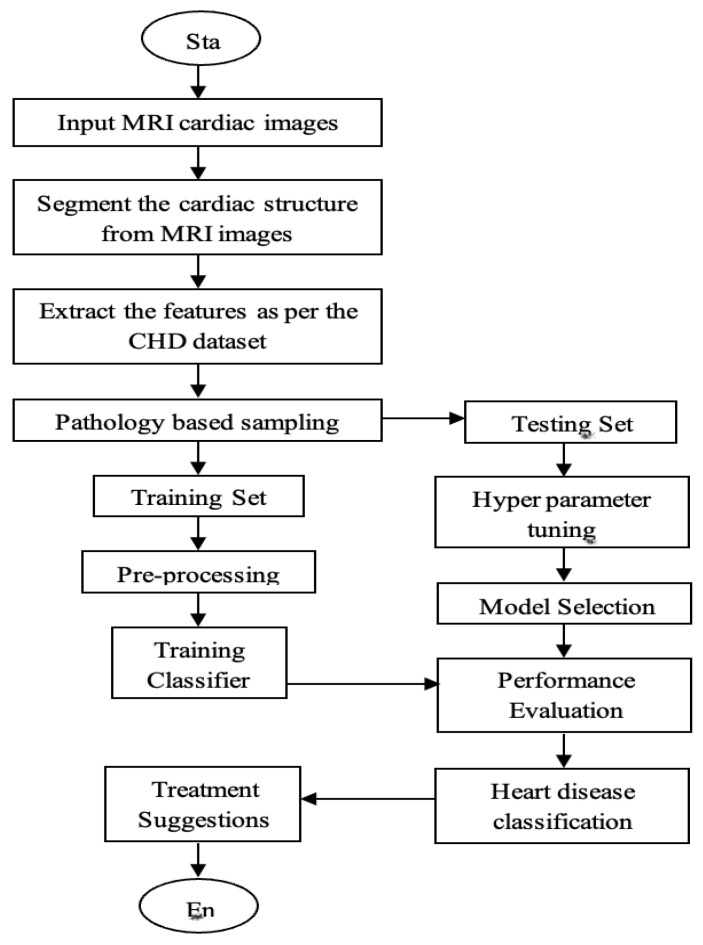
Proposed flow diagram.

The description for the above proposed flow diagram in Figure 2 is given below:

(1) MRI Image Input: The first step involves feeding MRI cardiac images into the CDLM system. These images provide detailed information about the structure and functioning of the heart.

(2) Cardiac structure segmentation: CDLM performs segmentation to accurately outline the boundaries of various cardiac structures in the MRI images. This ensures that only relevant regions of the heart are considered for further analysis.

(3) Feature extraction: Once the cardiac structure is segmented, the CDLM system extracts relevant features from the segmented regions. These features could include shape, size, texture, and other measurable characteristics that provide insights into CHD risk factors.

(4) CHD Dataset and Pathology-based Sampling: CDLM utilizes a CHD dataset containing information about individuals with CHD and their clinical outcomes. Pathology-based sampling is employed to select appropriate samples from the dataset, representing a diverse range of CHD cases and outcomes.

(5) Hyperparameter Tuning: CDLM optimizes its performance by adjusting hyperparameters such as learning rate, batch size, and network architecture. This fine-tuning process aims to improve the model accuracy and generalization capabilities.

(6) Heart Disease Classification: The CDLM model employs deep learning techniques to classify heart disease based on the extracted features and the selected CHD dataset. It learns patterns and relationships to determine the risk factors associated with CHD mortality.

(7) Treatment Suggestion: Based on the classification results, the CDLM system provides treatment suggestions or recommendations. These may include personalized treatment plans, lifestyle modifications, or interventions to mitigate the identified risk factors.

## 5. Analytical Discussion

In recent years, DL has emerged as a powerful tool for predictive modeling, providing state-of-the-art results in various domains. In this work, we explore the use of DL to predict newborn mortality in CHD. Congenital anatomical defects develop in the womb. In total, 6–8 babies out of 1000 newborns are born with defects. Even if the pregnancy usually continues and all the necessary tests are passed, the baby should be carefully examined after birth. Signs and symptoms of congenital heart defects in young children that can alert the child’s parents include:

- Heart murmur: The doctor may hear a characteristic sound when listening to the baby’s heart. In this case, echocardiography should be performed to exclude the defect.
- Weight gain: If the baby receives enough nutrition in the first months of life, but the weight gain does not exceed 400 g, it is worth arranging an appointment with a pediatrician.
- Shortness of breath: Fatigue may occur during feeding; the child eats a little, but most of the time. A pediatrician should address shortness of breath, and a referral to a cardiologist should be arranged.
- Tachycardia: On follow-up testing, the doctor may detect a rapid heartbeat.
- Cyanosis: The baby’s lips, heels, and fingertips turn blue. This may indicate a lack of oxygen in the blood due to a defect in the cardiovascular system.

The LaGrange function for CHD detection is considered with the different segment parts of the MRI images. These are all expressed in Equation (1).

(1) $$f\left(a\right)=sign\left(\mu a+e\right),\;$$

where *a* indicates the accurate identification of region in MRI input images, µ is the hyperplane normalized weight vector. The value of *µ* is obtained in Equation (2).

(2) $$\mu =\sum_{i=1}^{\infty }a_{i}*b_{i}*z_{i}.\;$$

Meanwhile, the values of *e* can obtained as the following Equations (3) and (4).

(3) $$e=b_{i}-\mu^{q}*a_{i},\;$$

(4) $$b_{i}(\mu^{q}*a_{i}+e)\geq 1;i=1,2,\ldots ,N;.$$

Normalization error is minimized, and we the following Equation (5) is obtained:

(5) $$W(\mu ,e,a)=\frac{1}{2}\mu^{q}*\mu *b_{i}(\mu^{q}*a_{i}+e)\leq 1;i=1,2\ldots ,N;,$$

(6) $$W(\mu ,e,a)=\frac{1}{2}\mu^{q}*\mu +\sum_{i=1}a_{i}\left(1-b_{i}*\mu^{q}e_{i}\right).$$

By including the stack vector value μ in the Equation (6), we can obtain the acute cardiac arrest details:

(7) $$b_{i}(\mu^{q}*a_{i}+e)\geq 1-\beta ;i=1,2,\ldots ,N;$$

Hence, we obtain the received prediction in Equation (8).

(8) $$A\left(a_{i},a_{j}\right)=\exp\left(-\frac{a_{i}-a_{j}{}^{2}}{2\mu^{2}}\right)=\exp\left(-\beta a_{i}-a_{j}{}^{2}\right),$$

(9) $$e=b_{i}-\sum_{j}\alpha_{j}b_{j}X\left(a_{j},a_{i}\right)\forall i;\alpha_{i}>0,$$

(10) $$\sum_{i}e_{i}-\frac{1}{2}\sum_{i,j}e_{i}e_{j}b_{i}b_{j}X\left(a_{i},a_{j}\right)s.t.\sum_{i}\alpha_{i}b_{i}=0\prime \;.$$

The binary classification issues are resolved with the following Equation (11):

(11) $$\mu^{T}a+e=\sum_{i}\alpha_{i}b_{i}X\left(a_{i},a\right)+e.$$

There is much potential for DL models to help improve outcomes for newborns with CHD. One promising area is using these models to predict mortality due to this condition. Currently, there are several limitations to using DL models for this purpose. More data on CHD need to be collected, which currently makes it challenging to train effective models. Most existing data are retrospective and may not represent the general population of newborns with this condition. The DL models require much data to be effective, and collecting enough data on individual patients with CHD is often tricky. Despite these limitations, DL models promise to improve outcomes for newborns with CHD. With more data and better algorithms, these models could be used to predict mortality due to this condition and help guide decisions about treatment.

## 6. Comparative Analysis

A comparative analysis of CHD prediction models should analyze the performance of different models for predicting CHD in a given population. This can be achieved by comparing each model’s accuracy, sensitivity, specificity, positive predictive value, and negative predictive value, as well as any other relevant performance metrics. The comparative analysis should also consider the cost of each model, the time required to develop and implement the model, and the ease of use or complexity. Finally, the analysis should consider any other factors that might be important to consider, such as the availability of data to train and test the models, the generalizability of the results, and the scalability of the model. The proposed cardiac DL model (CDLM) has been compared with the existing Novel healthcare framework (NHF), Decision Support System for Early Prediction (DSSEP), Machine Learning-Based Discharge Prediction (MLBDP), and Predictive Analysis of Congenital Heart Defects (PACHD). Here, the Matlab r2022a is the simulation tool used to execute the results.

### 6.1. Computation of Sensitivity (S_e_)

The sensitivity of CHD prediction is the ability of a test or procedure to identify those with the disease accurately. It is usually expressed as a percentage and is calculated by dividing the number of true positives by the total number of people with the disease. A higher sensitivity indicates a more accurate test or procedure. The computation of sensitivity is shown in the following Equation (12),

(12) $$S_{e}=\left(\frac{T_{p}}{T_{p}+F_{n}}\right),\;$$

where *S_e_* indicates the sensitivity of the CHD prediction, *T_p_* represents the optimistic accurate prediction, and *F_n_* indicates the pessimistic false prediction. Table 2 expresses the sensitivity Evaluation between the existing Novel healthcare framework (NHF), Decision Support System for Early Prediction (DSSEP), Machine Learning Based Discharge Prediction (MLBDP), Predictive Analysis of Congenital Heart Defects (PACHD) and the proposed cardiac DL model (CDLM).

**Figure 3:**
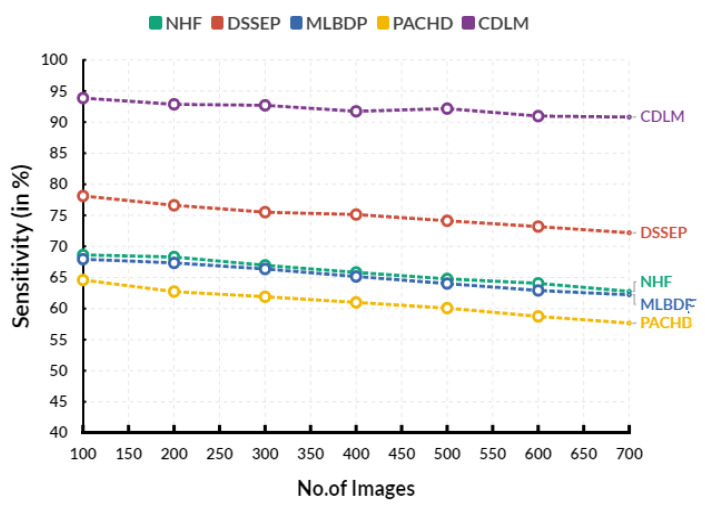
Evaluation of Sensitivity.

Figure 3 shows the Evaluation of sensitivity in various input images. In an Evaluation circle, the existing Novel healthcare framework (NHF) reaches 65.82%, Decision Support System for Early Prediction (DSSEP) obtains 75.13%, Machine Learning Based Discharge Prediction (MLBDP) reaches 65.16%, and Predictive Analysis of Congenital Heart Defects (PACHD) obtains 60.99% of sensitivity. The proposed cardiac DL model (CDLM) achieves 91.74% sensitivity. There is a great deal of sensitivity when it comes to predicting CHD. The condition can be caused by several factors, many of which are not yet fully understood. It means that even the most experienced medical professionals sometimes struggle to accurately determine diagnosis. It can be highly frustrating for parents worried about their child’s health. It is important to remember, however, that even though the prediction may not be 100% accurate, it can still help doctors determine what to look for and ways to treat the condition.

### 6.2. Computation of Specificity (S_p_)

The specificity of CHD prediction is the probability that a positive test result correctly indicates a CHD’s presence. It is typically expressed as a percentage and is calculated by dividing the number of accurate positive results by the total number of positive results. The computation of specificity is shown in the following Equation (13):

(13) $$S_{p}=\left(\frac{T_{n}}{T_{n}+F_{p}}\right),$$

where *S_p_* indicates the specificity of the CHD prediction, *T_n_* represents the true pessimistic prediction, and *F_p_* indicates the optimistic false prediction. Table 3 expresses the specificity evaluation between the existing Novel healthcare framework (NHF), Decision Support System for Early Prediction (DSSEP), Machine Learning-Based Discharge Prediction (MLBDP), Predictive Analysis of Congenital Heart Defects (PACHD) and the proposed cardiac DL model (CDLM).

**Figure 4:**
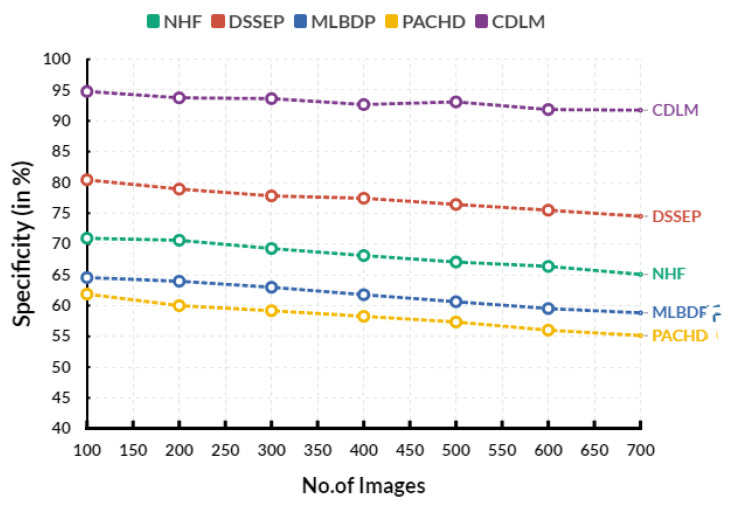
Evaluation of specificity.

Figure 4 shows the evaluation of specificity in various input images. In an evaluation circle, the existing Novel healthcare framework (NHF) reaches 68.12%, Decision Support System for Early Prediction (DSSEP) obtains 77.43%, Machine Learning Based Discharge Prediction (MLBDP) reaches 61.76%, and Predictive Analysis of Congenital Heart Defects (PACHD) obtains 58.25% of specificity. The proposed cardiac DL model (CDLM) achieves 92.65% specificity. The specificity of CHD prediction refers to the ability to accurately identify individuals who do not have the condition. It is typically measured in terms of the percentage of false-positive results or the number of individuals incorrectly identified as having the condition when they do not. Higher specificity is desirable for diagnostic testing, as it reduces the risk of misdiagnosis or unnecessary treatments. Specificity can be improved by using more advanced techniques, such as genetic testing, imaging techniques, or biomarker analysis.

### 6.3. Computation of Positive Prediction Value (PPV)

The Positive prediction value (PPV) measures the accuracy of a test to identify those with a disease correctly. It is calculated by dividing the number of accurate positive tests (correctly identified as having the disease) by the total number of positive tests (true and false positives). In the case of CHD prediction, PPV would be the proportion of correctly identified cases of CHD divided by the total number of cases predicted to have CHD. The computation of the positive predictive value is shown in the following Equation (14):

(14) $$PPV=\left(\frac{T_{p}}{T_{p}+F_{p}}\right),$$

where the *PPV* indicates the positive predictive value of the CHD prediction, *T_p_* represents the optimistic accurate prediction, and *F_p_* indicates the false positive prediction. Table 4 expresses the positive prediction value evaluation between the existing Novel healthcare framework (NHF), Decision Support System for Early Prediction (DSSEP), Machine Learning Based Discharge Prediction (MLBDP), Predictive Analysis of Congenital Heart Defects (PACHD) and the proposed cardiac DL model (CDLM).

**Figure 5:**
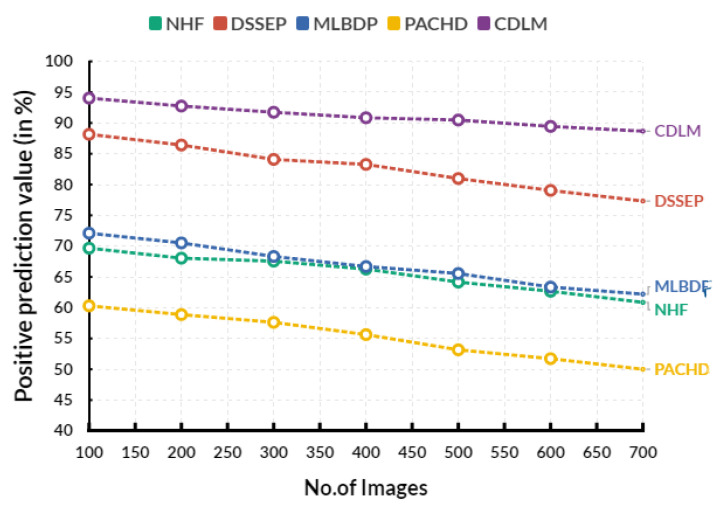
Evaluation of positive prediction value.

Figure 5 shows the evaluation of positive predictive value in various input images. In an evaluation circle, the existing Novel healthcare framework (NHF) reaches 66.27%, Decision Support System for Early Prediction (DSSEP) obtains 83.27%, Machine Learning Based Discharge Prediction (MLBDP) reaches 66.69%, and Predictive Analysis of Congenital Heart Defects (PACHD) obtains 55.63% of positive predictive value. The proposed cardiac DL model (CDLM) achieves a 90.85% positive prediction value. The optimistic prediction value (PPV) of CHD prediction is the proportion of patients with a positive test result for CHD correctly identified. It is calculated as the number of true positives divided by the sum of true and false positives. A higher PPV indicates a higher accuracy in the test used to predict CHD.

### 6.4. Computation of Negative Prediction Value (NPV)

The pessimistic prediction value of CHD prediction is the probability that a person does not have CHD, given that a test result is negative. It is calculated by dividing the number of true negatives (people who do not have the disease and whose test results are negative) by the total number of people who tested negative. The computation of the negative prediction value is shown in the following Equation (15):

(15) $$NPV=\left(\frac{T_{n}}{T_{n}+F_{n}}\right),$$

where the *NPV* indicates the negative predictive value of the CHD prediction, *T_n_* represents the accurate pessimistic prediction, and *F_n_* indicates the false negative prediction. Table 5 expresses the negative prediction value evaluation between the existing Novel healthcare framework (NHF), Decision Support System for Early Prediction (DSSEP), Machine Learning Based Discharge Prediction (MLBDP), Predictive Analysis of Congenital Heart Defects (PACHD) and the proposed cardiac DL model (CDLM).

**Figure 6:**
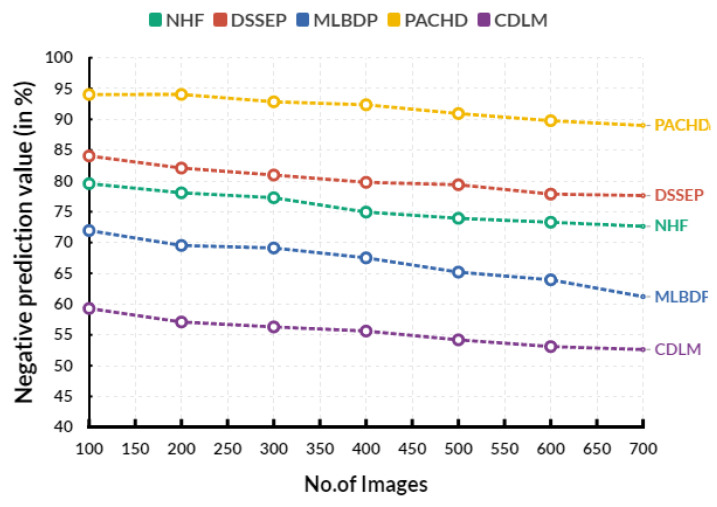
Evaluation of negative prediction value.

Figure 6 shows the evaluation of negative predictive value in various input images. In an evaluation circle, the existing Novel healthcare framework (NHF) reaches 74.94%, Decision Support System for Early Prediction (DSSEP) obtains 79.77%, Machine Learning Based Discharge Prediction (MLBDP) reaches 67.51%, and Predictive Analysis of Congenital Heart Defects (PACHD) obtains 92.37% of negative prediction value. The proposed cardiac DL model (CDLM) achieves a 55.62% negative prediction value. The pessimistic prediction value of CHD prediction is the percentage of patients who tested negative for the disease but were falsely diagnosed. This number is essential to understand how reliable the test is in accurately predicting the presence of the disease.

### 6.5. Computation of Miss Rate (R_m_)

The miss rate of CHD prediction is the percentage of instances where the prediction is incorrect. This rate is typically calculated by dividing the number of incorrect predictions by the total number of predictions made. The computation of the miss rate value is shown in the following Equation (16),

(16) $$R_{m}=\left(\frac{F_{n}}{F_{n}+T_{p}}\right).$$

where the *R_m_* indicates the miss rate value of the CHD prediction, *F_n_* represents the pessimistic false prediction, and *T_p_* indicates the optimistic accurate prediction. Table 6 expresses the miss rate Evaluation between the existing Novel healthcare framework (NHF), Decision Support System for Early Prediction (DSSEP), Machine Learning Based Discharge Prediction (MLBDP), Predictive Analysis of Congenital Heart Defects (PACHD) and the proposed cardiac DL model (CDLM).

**Figure 7:**
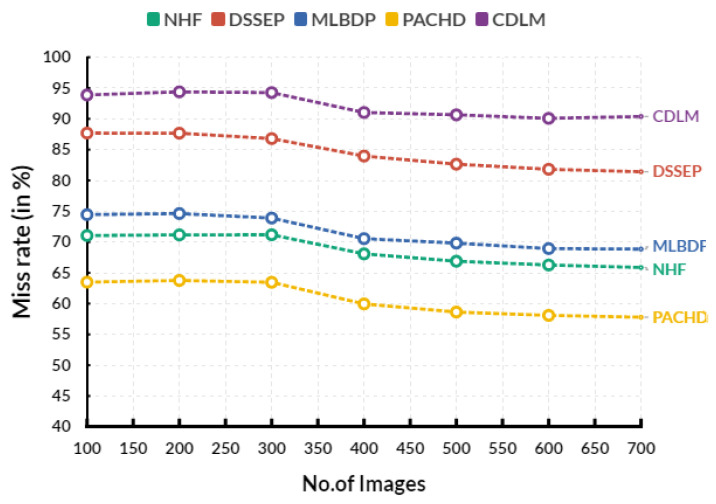
Evaluation of miss rate.

Figure 7 shows the evaluation of the miss rate in various input images. In an evaluation circle, the existing Novel healthcare framework (NHF) reaches 68.08%, Decision Support System for Early Prediction (DSSEP) obtains 83.96%, Machine Learning Based Discharge Prediction (MLBDP) reaches 70.55%, and Predictive Analysis of Congenital Heart Defects (PACHD) obtains59.95% of miss rate. The proposed cardiac DL model (CDLM) achieves a 91.03% of miss rate. The miss rate of CHD prediction measures how often a predicted diagnosis of CHD is incorrect. It is calculated by dividing the number of incorrect predictions by the total number of predictions made. The lower the miss rate, the more accurate the predictions are. Miss rates are typically expressed as a percentage. Table 7 shows the overall evaluation between the existing and the proposed models.

**Figure 8:**
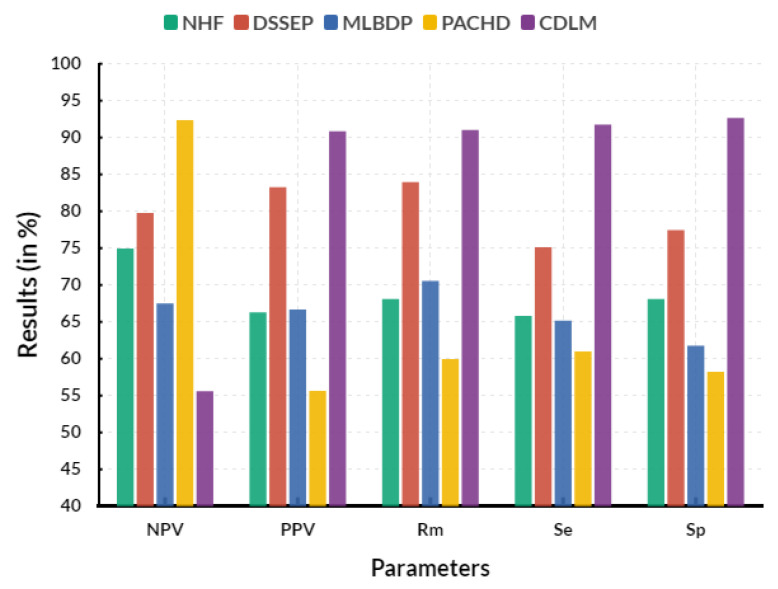
Overall Evaluation.

Figure 8 shows the overall evaluation between the existing and proposed models. In an Evaluation circle, the proposed cardiac DL model (CDLM) achieves 91.74% of sensitivity, 92.65% of specificity, 90.85% of positive prediction value, 55.62% of negative prediction value and 91.03% of miss rate. The results of comparative analysis can provide a comprehensive view of the genetic risk factors associated with CHD, allowing for a more accurate prediction of the disease. It can also reveal the potential for new treatments and uncover previously unknown genetic risk factors for the disease. The results of the comparative analysis allow for a greater understanding of the complex interaction between genes and environment, which can help to explain why specific individuals are more susceptible to developing the disease than others. It can also provide valuable insights into the biological pathways that lead to CHD’s development and help identify new targets for therapeutic interventions. It is a cost-effective tool for predicting and preventing CHD, as it can be performed quickly and easily with existing genetic data.

## 7. Conclusions

Congenital heart disease can also affect the valves that control blood flow through the heart or the arteries and veins that carry blood to and from the heart. Sometimes, the heart may be unable to pump enough blood to meet the body’s needs. CHD can be treated with surgery, medication, or both. In some cases, the heart may be able to repair itself. However, many babies with CHD need lifelong treatment. Machine learning can improve the accuracy of imaging techniques used to diagnose CHD. By training a machine learning model to detect the presence of CHD, healthcare providers can more accurately diagnose the condition and provide appropriate care. The proposed cardiac DL model (CDLM) achieves 91.74% of sensitivity, 92.65% of specificity, 90.85% of positive prediction value, 55.62% of negative prediction value and 91.03% of miss rate. Overall, machine learning has the potential to reduce newborn mortality suffering from CHD significantly. By developing models to identify risk factors associated with mortality and providing early detection and intervention, healthcare providers can ensure that high-risk infants receive the care they need to survive. In addition, the proposed machine learning can be used to develop predictive models that accurately identify newborns with CHD at risk of mortality. These models could then be used to alert healthcare providers of possible high-risk cases so that they can provide appropriate care early, thus potentially reducing mortality rates.

## Figures and Tables

**Table 1:** Comparative analysis.

Authors	Research Highlights
Edupuganti, M. et al. [21]	The acquired defects in children and adolescents are characterized by persistent changes in the structure of the heart. The initial changes are carried out after the birth of a child, causing a disorder in the functioning of the heart
Eltahir, M.M. et al. [22]	In clinical practice, acquired heart defects are classified in different ways. The white type of CHD is triggered by stimulation of the skin. It is characterized by the release of blood from an arterial circulation into a venous one
Katarya, R. et al. [23]	The blue-type defects are visually distinguished by cyanosis of the skin (persistent cyanosis) transfer of large vessels. It is caused by a process where the aorta leaves the right ventricle, and the pulmonary artery, on the contrary, proceeds from the left
Nadakinamani, R.G. et al. [24]	The Fallot triad is a combination of several disorders like narrowing of the pulmonary artery, ventricular septal defect, aortic and right ventricular defects. These include right vena cava atresia, pulmonary artery, aorta, etc.
Tan, W. et al. [25]	The Fetal echocardiography is a non-invasive method for diagnosing CHDs in fetuses. The machine learning approach using artificial neural networks has to minimize the risk of death due to CHD
Hussain, L. et al. [26]	The ANN prediction model was trained on medical data sets and tested for accuracy in predicting the outcome of the newborns suffering from CHD
Al Ahdal, A. et al. [27]	The diagnosis model enabled accurate disease diagnosis by leveraging data from history using machine learning techniques, leading to more accurate predictions on patient’s prognosis and treatment options
Dritsas, E. et al. [28]	The screening tools and predictive models are used to identify risk groups for newborns suffering from CHD. These tools allow for early detection, helping to identify those at higher risk and provide them with appropriate care
Ng, W. et al. [29]	Machine learning is also being used to develop a risk index which can help in the present analysis of pregnancy and predict the possibility of a newborn having CHD
Balakrishnan, M. et al. [30]	The machine learning algorithms such as logistic regression and decision trees can be used to analyze the data collected from maternal laboratory tests, clinical laboratory data, and other studies predicting CHD
Williams, R. et al. [31]	Through CHD prediction using machine learning techniques such as supervised learning, it is possible to develop predictive models that are able to accurately predict CHD in newborns
Ravi, R. et al. [32]	With data obtained through laboratory tests combined with machine learning algorithms such as logistic regression and decision trees, it is possible for doctors to create accurate predictive models
Pei, Y. et al. [33]	Integrated patient data from a great variety of sources can be used to create efficient machine learning models that use ML algorithms to identify at-risk newborns before their birth
Shishah, W. et al. [34]	The machine learning classification approach can be used to identify at-risk infants and diagnose them quickly, allowing doctors to take preventative measures early on
Iscra, K. et al. [35]	The machine learning technology has been utilized to develop predictive models for the diagnosis of newborns with CHD. These models have been applied to large datasets of neonatal ICU admissions and have shown promising results in terms of accuracy and speed of diagnostics

**Table 2:** Evaluation of sensitivity (in %).

No. of Images	NHF	DSSEP	MLBDP	PACHD	CDLM
100	68.63	78.12	67.94	64.60	93.87
200	68.30	76.62	67.35	62.73	92.86
300	66.96	75.51	66.37	61.90	92.70
400	65.82	75.13	65.16	60.99	91.74
500	64.77	74.12	64.02	60.07	92.17
600	64.06	73.19	62.91	58.74	90.97
700	62.76	72.19	62.21	57.66	90.81

**Table 3:** Evaluation of specificity (in %).

No. of Images	NHF	DSSEP	MLBDP	PACHD	CDLM
100	70.93	80.42	64.54	61.86	94.78
200	70.60	78.92	63.95	59.99	93.74
300	69.26	77.81	62.97	59.16	93.61
400	68.12	77.43	61.76	58.25	92.65
500	67.07	76.42	60.62	57.33	93.08
600	66.36	75.49	59.51	56.00	91.84
700	65.06	74.49	58.81	55.13	91.73

**Table 4:** Evaluation of positive prediction value (in %).

No. of Images	NHF	DSSEP	MLBDP	PACHD	CDLM
100	69.67	88.16	72.10	60.30	94.04
200	68.04	86.42	70.52	58.88	92.75
300	67.56	84.08	68.32	57.62	91.74
400	66.27	83.27	66.69	55.63	90.85
500	64.16	80.98	65.55	53.16	90.48
600	62.67	79.05	63.35	51.72	89.44
700	60.86	77.32	62.20	50.00	88.67

**Table 5:** Evaluation of negative prediction value (in %).

No. of Images	NHF	DSSEP	MLBDP	PACHD	CDLM
100	79.56	84.06	71.94	94.04	59.29
200	78.07	82.09	69.52	94.05	57.09
300	77.27	80.96	69.11	92.85	56.29
400	74.94	79.77	67.51	92.37	55.62
500	73.93	79.38	65.19	90.94	54.19
600	73.29	77.86	63.94	89.78	53.10
700	72.63	77.62	61.21	89.01	52.62

**Table 6:** Evaluation of miss rate (in %).

No. of Images	NHF	DSSEP	MLBDP	PACHD	CDLM
100	71.05	87.69	74.45	63.49	93.88
200	71.16	87.67	74.62	63.76	94.38
300	71.18	86.79	73.89	63.46	94.26
400	68.08	83.96	70.55	59.95	91.03
500	66.88	82.64	69.82	58.63	90.65
600	66.27	81.81	68.93	58.09	90.08
700	65.86	81.41	68.85	57.79	90.38

**Table 7:** Overall Evaluation.

Parameters	NHF	DSSEP	MLBDP	PACHD	CDLM
Sensitivity (*S_e_*)	65.82	75.13	65.16	60.99	91.74
Specificity (*S_p_*)	68.12	77.43	61.76	58.25	92.65
Positive prediction value (*PPV*)	66.27	83.27	66.69	55.63	90.85
Negative prediction value (*NPV*)	74.94	79.77	67.51	92.37	55.62
Miss rate (*Rm*)	68.08	83.96	70.55	59.95	91.03

## Data Availability

The data presented in this study are available in [insert article].
